# Organ agar serves as physiologically relevant alternative for *in vivo* colonization

**DOI:** 10.21203/rs.3.rs-2777869/v1

**Published:** 2023-05-19

**Authors:** Melanie M. Pearson, Allyson E. Shea, Sapna Pahil, Sara N. Smith, Valerie S. Forsyth, Harry L. T. Mobley

**Affiliations:** Department of Microbiology and Immunology, University of Michigan, Ann Arbor, Michigan, USA

## Abstract

Animal models for host-microbial interactions have proven valuable, yielding physiologically relevant data that may be otherwise difficult to obtain. Unfortunately, such models are lacking or nonexistent for many microbes. Here, we introduce organ agar, a straightforward method to enable the screening of large mutant libraries while avoiding physiological bottlenecks. We demonstrate that growth defects on organ agar were translatable to colonization deficiencies in a murine model. Specifically, we present a urinary tract infection agar model to interrogate an ordered library of *Proteus mirabilis* transposon mutants, with accurate prediction of bacterial genes critical for host colonization. Thus, we demonstrate the ability of *ex vivo* organ agar to reproduce *in vivo* deficiencies. This work provides a readily adoptable technique that is economical and uses substantially fewer animals. We anticipate this method will be useful for a wide variety of microorganisms, both pathogenic and commensal, in a diverse range of model host species.

## Introduction

Animal models have a longstanding track record in the study of microbial pathogens, including the fulfillment of Koch’s third postulate (1). Although an excellent model is indispensable for virulence studies, such models may be difficult to establish for fastidious or host-restricted pathogens. Furthermore, even when models exist, complex disease progression can confound experimental interpretation. For example, in the urinary tract, infecting microbes exhibit planktonic growth in urine, adhere or invade the bladder epithelium, ascend into the kidneys, and disseminate into the bloodstream (2, 3). Microbes compete with the host immune response and with established bladder microbiota (4, 5). Each of these challenges can manifest as a chokepoint, or bottleneck, where only a limited number of microbes become established. These bottlenecks have a profound effect on experimental design, particularly in modern large-scale studies, where the number of mutants that can be feasibly studied without random loss is limited due to such founder effects (6-9).

Urinary tract infections (UTIs) are one of the most common infections worldwide, affecting most women at least once in their lifetime (10, 11). These infections are even more common in patients with urinary catheters, where a more diverse mix of bacterial species is likely to be the causative agent. In particular, *Proteus mirabilis* is especially problematic in patients with long-term (>30 days) indwelling urinary catheters (12-14). This species produces urease, which cleaves urea, abundantly present in urine, into ammonia and carbon dioxide. The resulting pH increase causes precipitation of calcium and magnesium ions naturally present in urine, to form struvite and hydroxyapatite crystals that can block catheter flow and directly damage urinary tract tissue via stone formation (urolithiasis) (15).

Previous studies on *P. mirabilis* virulence have relied on a well-established murine model of UTI (15). Most often, this model involves instillation of bacteria directly into the bladder via a catheter, which can either be removed to study UTI progression in the absence of a foreign body, or fully pushed into the bladder to model long-term catheterization (16-18). In either case, *P. mirabilis* readily establishes infection and, similar to human UTI, may cause pyelonephritis and urolithiasis (15). In addition to challenging mice with one or two strains at a time, the mouse model has been used to screen pools of mutants from uropathogenic species of bacteria (6, 19).

We aimed to conduct similar large-scale studies to identify genes that contribute to UTI in mice. Toward this goal, we present data showing the experimental bottleneck for the total number of *P. mirabilis* mutants that can be tested in this model without stochastic loss is much narrower than anticipated. We recently built an ordered library containing single transposon insertions in 1728 genes (20). Based on the tight experimental bottleneck, we concluded that screening this library in a murine model would not be feasible. Thus, we devised a method to assay the ordered library on agar made by mixing organ homogenates with molten agar to create “organ agar.” In this study, we show that organ agar, coupled with agar made from pooled human urine, identified genes that contribute to *P. mirabilis* fitness during UTI. Many of these genes were part of biosynthetic pathways, indicating that organ agar is especially useful for identifying nutritional availability in distinct organ sites. Excitingly, most (6 of 7) of the mutants we selected for follow-up study were significantly impaired when tested in mouse model cochallenges. We anticipate that this economical model will open new avenues of research for many diverse pathogens.

## Results

### The bottleneck effect for *Proteus mirabilis* in a murine model of UTI is much stronger than previously described.

**Figure 1.**
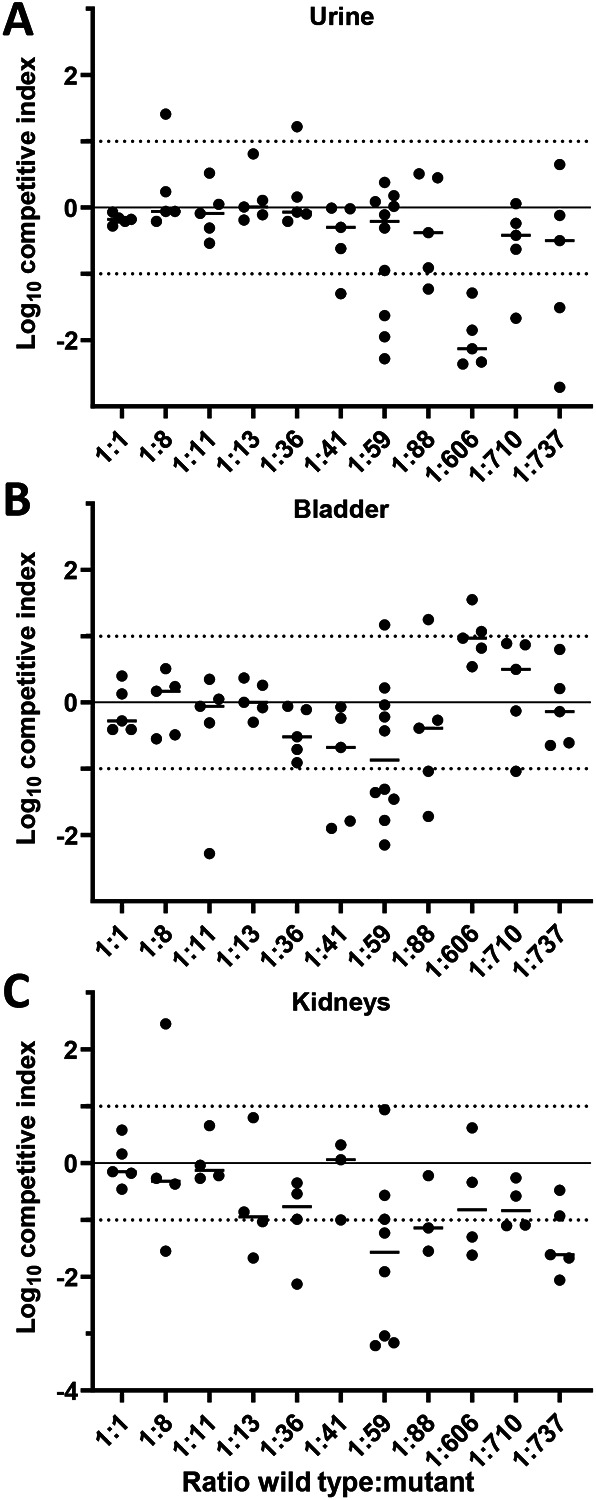
Determining the bottleneck of infection for *P. mirabilis* during UTI. (A-C) CBA/J mice were transurethrally inoculated with 10^7^ CFU. The inoculum contained different ratios of wild-type HI4320 and marked mutant *spa47* (kan^R^) that was previously determined to have no fitness defect (ref). The ratios are indicated on the *x*-axis. At 24 h post-inoculation, (A) urine, (B) bladder, and (C) kidneys were harvested. Each sample was subjected to differential plating to enumerate the ratio of wild-type to *spa47* in the organ. A competitive index (CI) was calculated and is plotted on the *y*-axis. Each dot represents a single mouse (n=5-10); bars indicate the median. A log CI of 0 indicates that the wild-type and mutant were recovered in the same ratio as were introduced in the inoculum. Dotted lines at ± 1 indicate the acceptable maximum variation for this experiment.

Our group and others have successfully enumerated genetic bottleneck effects observed in the traditional model of ascending UTI (6, 21, 22). Although relatively generous infection dynamics of *P. mirabilis* in mice have been estimated for a murine model where a catheter segment is entirely inserted into the bladder lumen (18, 19), we found the bottleneck for *P. mirabilis* is narrow in the traditional UTI model and would therefore be problematic for large screening experiments *in vivo* (Fig. 1). After testing different input ratios of a non-deleterious mutant and wild type in cochallenges, we determined that only very small groups of strains can be assembled to avoid stochastic loss of mutants. In urine, up to 100 strains could be tested with the ratio remaining stable throughout the infection, calculated as a log competitive index (CI) of zero (Fig. 1A). However, even at ratios as low as 1:8, we observed 1/5 mice displaying results outside of the ideal range, indicating stochastic loss of the mutant. Bladder samples became unacceptable at an even lower ratio of 1:59, where over half of mice fell outside the preferred range (Fig. 1B). Around 20% of mice with colonization in the urine and bladder had no detectable CFU burden in the kidneys (Table 1). This, in combination with the increased spread of data between biological replicates, caused cochallenge input ratios as low as 1:13 to be problematic (Fig. 1C). Collectively, the bottleneck during murine UTI with *P. mirabilis* is severe and therefore limits the feasibility of *in vivo* experimental screens. In response, we sought alternative approaches for identifying genes for further study.

### Development of organ and urine agar.

Our goal was to test the fitness of an ordered transposon library built for *P. mirabilis* strain HI4320 (20) in a physiologically relevant condition. Because of the severe bottleneck in mice, we devised a screen that did not require pooling of mutants. Mouse bladders, kidneys, and spleens were aseptically collected to represent different stages of ascending UTI (cystitis, pyelonephritis, and bacteremia/urosepsis, respectively). Organs were homogenized in water to prevent salt-induced swarming motility by *P. mirabilis* (23, 24). These homogenates were either mixed 1:1 with autoclaved agar, or further diluted before mixing with agar. All organs sustained growth of *P. mirabilis*; however, growth on spleens yielded tiny colonies at 24h and colonies on bladder agar required at least 36h of incubation to be readily visible. In contrast, the larger and denser kidneys could be diluted up to 1:10 and still sustain growth (Supplemental Fig. S1). Uninoculated plates showed no outgrowth of microbiota during the duration of the experiment. We selected this 1:10 dilution for screening the ordered library on agar made from kidneys and switched spleens for larger livers to represent disseminated infection, which allowed us to screen 1728 single gene insertions using organs pooled from only five mice.

Similarly, agar made from pooled human urine was adapted for *P. mirabilis* using two modifications. First, the agar was buffered to counteract growth-inhibitory increases in pH due to ammonia released from urease activity (Supplemental Fig. S2A). Second, the agar concentration was increased to reduce swarming motility. This did not completely eliminate swarming but was sufficient for genetic screening (Supplemental Fig. S2B).

### Screening of *P. mirabilis* ordered library on organ and urine agar.

**Fig. 2.**
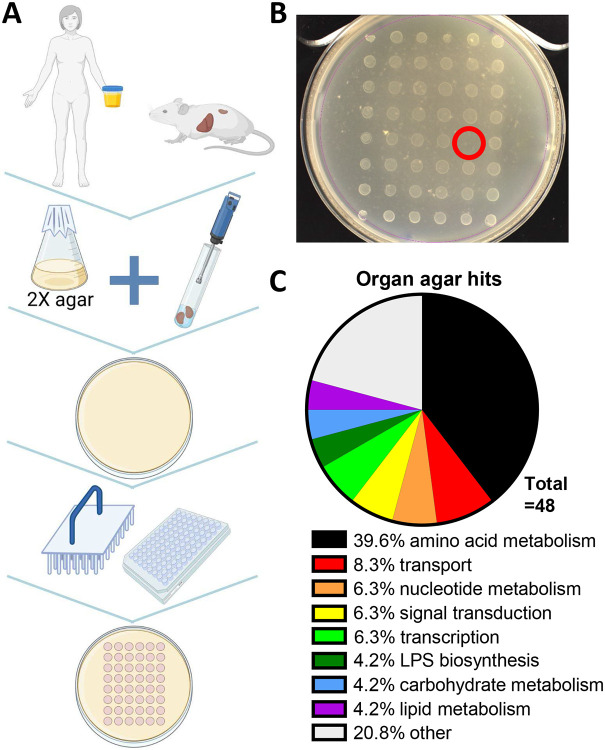
Organ agar screen. (A) Schematic for generating organ agar and library screen. (B) 1:10 kidney agar with 48 stamped library mutants. One transposon hit (lack of growth) is circled in red. (C) Classification of organ agar hits obtained on one or more agars. Genes classified as “other” include singly-binned genes with predicted functions and hypothetical genes.

Mutants in the ordered library were stamped onto liver, kidney, and urine plates and incubated at 37°C, and growth was recorded after 24 and 48 h (Fig. 2; Supplemental Fig. S3). A total of 48 mutants were qualitatively identified that had growth defects on one or more organ agars (Table 2). The largest category of hits was biosynthetic genes, particularly amino acids (Fig. 2C). We found genes known to contribute to *P. mirabilis* fitness during UTI (*i.e.*, high-affinity zinc uptake genes *znuA* and *znuC*) (25), as well as pathways known to be important (*e.g.*, TCA cycle) (26). We also identified known contributors to uropathogenic *Escherichia coli* (UPEC)-mediated UTI (*e.g.*, purines and branched-chain amino acid biosynthesis) (6).

### Selection of candidates for further study.

We selected 20 mutant hits for confirmation of the predicted transposon insertion using PCR. Consistent with our previous randomized testing of this library (20), 16 of 20 mutants were confirmed to have the predicted insertion (Supplemental Table S1 and Supplemental Fig. S4). We selected seven genes to validate with targeted mutagenesis (Table 2). These genes represent the major categories of genes from Fig. 2C (amino acid metabolism and purine biosynthesis) and mutants with phenotypes on either a single or all three types of agar (urine, kidney, or liver).

### Most organ agar mutants had nutritional deficiencies *in vitro*.

**Fig. 3.**
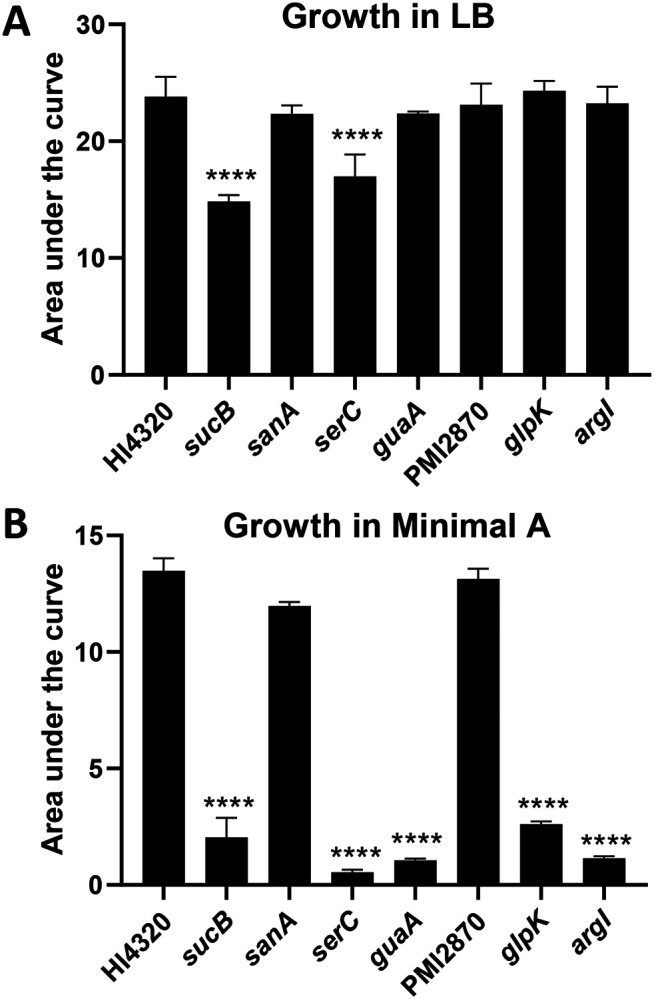
*P. mirabilis* wild type and mutant strains were assayed for growth in **(A)** LB (n = 3-6) or **(B**) chemically defined medium Minimal A (n = 4-5). Area under the curve was measured after culturing for 20 h in the indicated medium. Most mutants grew well in LB, which was the medium used for obtaining transposon mutants. However, most mutants had a deficiency in Minimal A. Error bars depict SD. Statistical significance was calculated *vs*. wild type using one-way ANOVA with Dunnett’s multiple comparisons test.

**Fig. 4.**
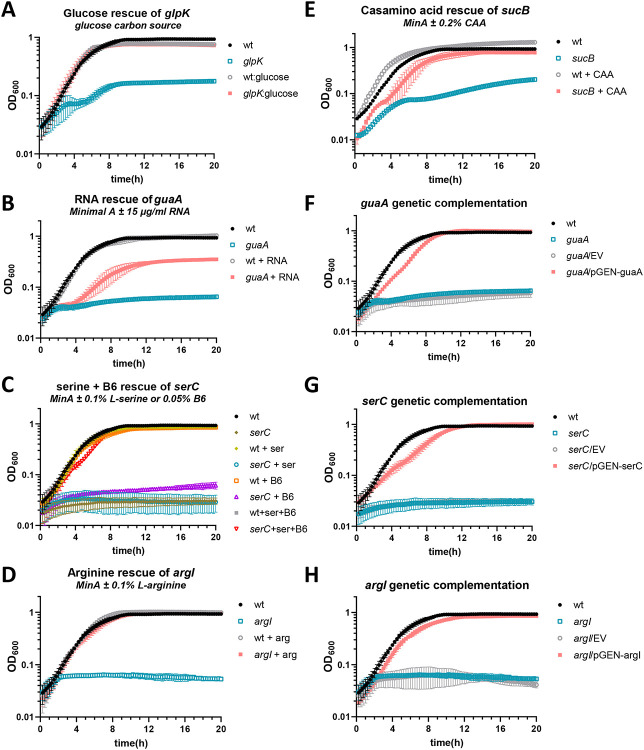
Chemical and genetic complementation is shown for a selection of mutants. For all growth curves, Minimal A medium with 0.2% glycerol as a carbon source was used as the base medium unless otherwise specified. (A-E) Chemical complementation growth curves. (A) Complementation of *glpK* was achieved by swapping the carbon source to 0.2% glucose. (F-H) Genetic complementation growth curves. MinA, Minimal A; B6, pyridoxine HCl; CAA, casamino acids; EV, empty vector (pGEN-MCS). n = 3-5 independent experiments for each condition. Error bars show SD.

We examined growth of all seven of the reconstructed mutants in a variety of media. Most exhibited robust growth in complex LB medium, as expected, because the transposon mutants were originally produced using this medium. The exceptions were *sucB*, which grew at a similar rate as wild type but saturated at a lower density, and *serC*, which grew more slowly but reached the same saturation as wild type (Fig. 3A and Supplemental Fig. S5A). However, in the chemically defined minimal medium Minimal A, most mutants (5 of 7) had profoundly diminished growth (Fig. 3B and Supplemental Fig. S4B). Only two, *sanA* and PMI2870, displayed normal growth. Based on the predicted function of each mutated gene, we supplemented Minimal A with appropriate substrates and rescued growth of all mutants. Thus, *glpK* mutant was rescued by swapping the carbon source from glycerol to glucose, *argI* was rescued by addition of L-arginine, *serC* was rescued with a combination of L-serine and vitamin B6 (27), *guaA* was rescued by the addition of purified *P. mirabilis* RNA, and *sucB* was rescued by the addition of casamino acids (Fig. 4A-E).

We selected three of these mutants for genetic complementation (*argI, serC*, and *guaA*). The first two are standalone genes and were cloned as a single fragment with their native promoters. The last gene, *guaA*, follows *guaB* as part of an operon (*guaBA*). This gene was cloned with the *gua* promoter, omitting *guaB*. In all three cases, genetic complementation restored growth in Minimal A (Fig. 4F-H).

#### Some mutants had altered swarming motility.

**Fig. 5.**
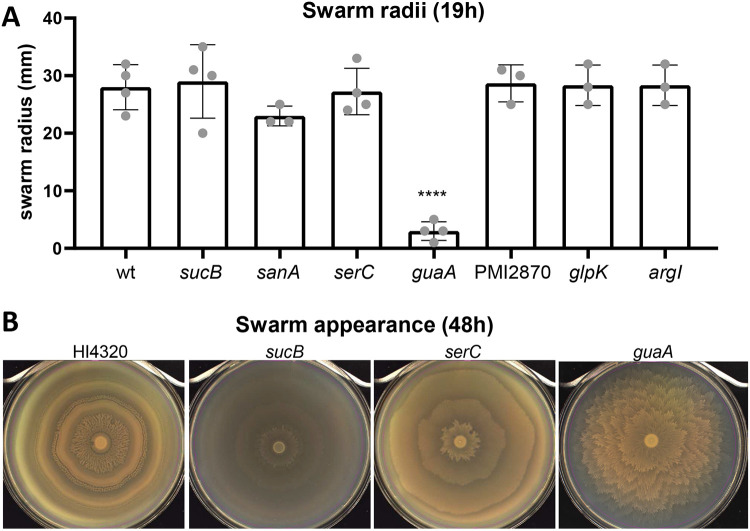
*P. mirabilis* readily swarms in a bullseye pattern on LB agar containing 10g/L NaCl. **(A)** Quantification of swarm radii after 19h at 30°C, before wild-type swarms reached the edge of the agar surface. Error bars show SD. Statistical significance was calculated *vs*. wild type using one-way ANOVA with Dunnett’s multiple comparisons test. **(B)** Photos of selected swarms after 48h incubation at 30°C, when swarming and consolidation rings are more visible. 3 of 7 of the organ agar hits had strikingly different patterns compared with wild type.

*P. mirabilis* readily colonizes surfaces via swarming motility, including catheters. Because genes involved in swarming often correlate with *in vivo* fitness defects (28, 29), we next assessed swarming motility by these mutants. When assayed for distance swarmed after 19 h of incubation, only the *guaA* mutant had a significant defect (Fig. 5A). However, *guaA* and two additional mutants, *serC* and *sucB*, exhibited swarming patterns strikingly distinct from the classic wild-type bullseye. These patterns were especially apparent with 48 h incubation time, allowing natural pigmentation to develop (Fig. 5B, Supplemental **Video S1**). Swarming by all seven mutants after either 19 h or 48 h incubation is shown in Supplemental Fig. S6.

#### Organ agar results were reproducible with other mice.

The initial organ agar screen was conducted with transposon mutants using outbred male Swiss Webster mice, selected for low cost and wide availability. We next examined agar made from our standard UTI model strain, female CBA/J mice, with the reconstructed mutants and found comparable results (Supplemental Fig. S7A). Notably, most phenotypes observed on diluted organs were also seen on agar made from undiluted homogenates, and we genetically complemented the growth defects for *guaA, serC*, and *argI*. Interestingly, complementation of *argI* led to a hyperaggressive swarming phenotype on urine agar (Supplemental Fig. S7B), consistent with previous observations that arginine is a swarming cue (30, 31). The major exception was PMI2870, which had a profound defect on all three agar types in the initial screen but no observable defect in the rescreen using CBA/J mice. Because the original screen involved stamping of samples from frozen plates and the rescreen used fresh broth cultures, we reasoned the difference could have resulted from the number of bacteria deposited on the plate. Accordingly, there was a growth defect observed for the PMI2870 mutant on liver agar after 100-fold dilution (Supplemental Fig. S7C).

Uropathogenic *E. coli* (UPEC) is the most common causative agent of uncomplicated UTI (3). To investigate whether our *P. mirabilis* findings extended to this species, we next tested whether UPEC mutants had similar deficiencies on organ agar. Homologous mutations in UPEC CFT073 were available for 5 of 7 of the target genes. Interestingly, all five of these mutants grew on all organ agars (Supplemental Fig. S7A). This is consistent with prior work where these mutants had no apparent defect in either human urine *ex vivo* or in murine bladders (6). Notably, UPEC often encodes redundancy that is not seen for *P. mirabilis* (26, 32-35).

#### Organ agar hits displayed defects in murine ascending UTI model.

**Fig. 6.**
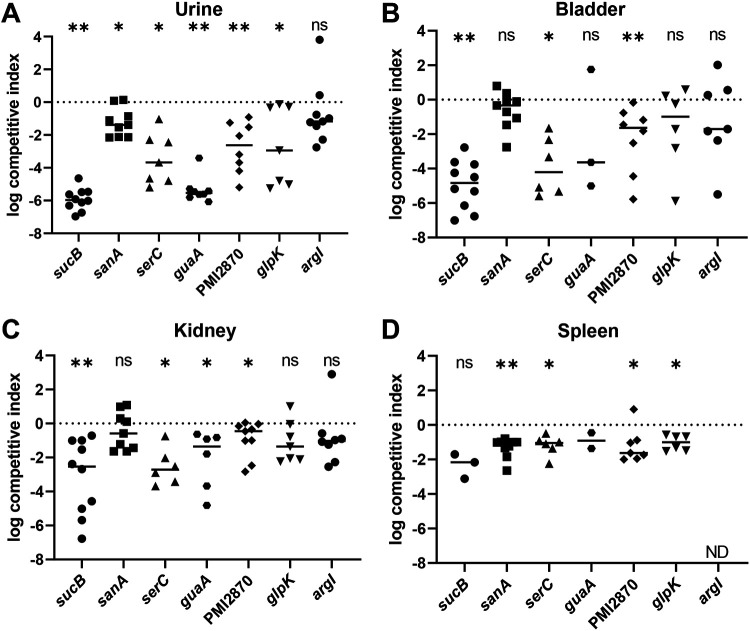
Female CBA/J mice were transurethrally inoculated with a 1:1 mixture of HI4320 (wt) and the indicated mutant. After 7 days, bacteria were quantitatively cultured and competitive indices were calculated. Each symbol represents an individual mouse. *P* values were assessed using the Wilcoxon signed rank test (*P<0.05; **P<0.01; ns, not significant). ND, not determined; a competitive index could not be determined because no bacteria were recovered.

We were especially interested to see if poor growth of mutants on organ agar translated to deficiencies in establishing UTI. To this end, we inoculated bladders of female CBA/J mice with a 1:1 mixture of wild-type *P. mirabilis* HI4320 and each mutant. After 7 days, urine was collected, then mice were sacrificed and bladders, kidneys, and spleens were removed. For 6 of the 7 mutants, there was a statistically significant defect in colonization compared with wild type in one or more of the assayed sites (Fig. 6). In three cases (*sucB, serC*, and *guaA*), the mutant was almost completely outcompeted by wild type. The exception was *argI*, which was outcompeted in urine, bladder, and kidneys by a median of 1-2 logs but was not statistically significant. Notably, the overall bacterial burden at 7 days post-inoculation was lower than usual in the *argI* cochallenge (Supplemental Fig. S8). In summary, lack of growth on organ agar was a highly effective predictor of fitness loss in the urinary tract of mice.

## Discussion

Microbiologists have historically cultured bacteria using media that are reflective of their origins. For example, soil organisms would use soil medium and marine organisms would use seawater (36, 37). Organ-derived media have been used since the inception of bacteriological culture to study mammalian pathogens, sometimes as a method to distinguish related bacterial species (38), but not to our knowledge as a method to screen mutant libraries for host fitness.

*P. mirabilis*, like many other bacterial species, colonizes and infects multiple sites within the human body to cause disease. Specifically, Proteus can colonize the gut, skin wounds, and the urinary tract (39, 40). These unique host niches are studied using various *in vivo* models, each with their own limitations (16, 19, 41-43). Here, we demonstrated that the urinary tract has strict bottleneck effects in the murine model, requiring the use of many animals for testing mutant libraries. To address this, we present an alternative screening method by generating agar from whole organ samples, greatly reducing the number of mice required for experimentation while increasing feasibility of large screens in models exhibiting limitations.

We screened 1728 individual *P. mirabilis* transposon mutants from an ordered transposon library (20) on human urine and murine kidney and liver agar. Stamping arrayed mutants onto agar only required 5 mice to screen this library. Using a calculated bottleneck of 25 mutants, a comparable Tn-seq experiment would have required 70 mice. In addition to being more ethically and fiscally responsible, this alternative *ex vivo* model strongly correlated with colonization defects *in* vivo, and thus serves as a proxy for more complex model systems. Indeed, 6 out of 7 mutants had statistically significant defects in either the urine, bladder, kidneys, or spleen of mice when examined in the traditional cochallenge model of ascending UTI (Fig. 6) and other validating hits were observed, such as zinc transporter genes *znuA* and *znuC* (25).

Our data show that organ agar is a physiologically relevant medium for testing colonization and virulence factors. However, certain variables may not be modeled as they are *In vivo*. For example, neutrophil recruitment plays an important role in *Proteus* uropathogenesis (44, 45). These non-resident immune cells would likely be in low numbers in the organs of naive mice, and those that are present in organ agar would not exhibit antimicrobial activities such as phagocytosis. Likewise, the architecture of different cell types is disrupted during homogenization. In the uroepithelium, for example, the surface-expressed residues used for bacterial adherence may be heterogeneously distributed throughout the agar. Deeper tissues such as the lamina and muscularis propria, which likely do not directly encounter the bacteria, are exposed in organ agar. Despite these potential pitfalls, organ agar detected fitness factors in functional categories including metabolism, transport, LPS biosynthesis, and even those with unpredicted function.

Most detected hits, 54.3%, fell into a metabolism-related category, suggesting that this method is particularly powerful for identifying the nutritional requirements of microorganisms in their respective host environments. Five of the 7 mutants selected for further follow-up analysis were part of well-studied metabolic pathways. Although the lack of growth of most mutants in minimal medium was initially surprising, several mutants exhibiting no growth defects in LB or minimal medium were outcompeted *in vivo*, demonstrating the relevance and sensitivity of *ex vivo* organ agar for discovering new fitness factors beyond metabolism. These findings contrast with a UPEC Tn-seq study, where a much smaller percentage (8%) of detected fitness factors were identified as metabolic (6). Yet, tailored metabolism is increasingly recognized as an integral part of UPEC virulence (46). The *E. coli* accessory genome is remarkably broad (47), and organ agar would greatly facilitate screening a wide variety of UPEC strains for nutritional fitness.

Organ agar reflects distinct niches; for example, liver and kidney give different results. We speculate that use of different host genetic backgrounds will also identify unique fitness determinates. For example, knockout mice may change the nutritional landscape for microorganisms. Similarly, urine agar could be made using samples from volunteers with specific diets or disease states.

Overall, we demonstrate that *ex vivo* organ agar is a reliable, sensitive method to predict fitness factors in model organisms that can be recapitulated *in vivo*. This is especially important for microorganisms that lack well-developed animal models. Additionally, organ agar can utilize animals that would normally be euthanized to maintain breeding colonies. Using different host models with unique genetic backgrounds, such as knockout mice, may yield surprising and exciting results and mitigate previously mentioned limitations such as immune interactions. We propose that additional refinements of the technique, such as improving plate clarity to allow quantitative densitometry measurements, will further expand the utility of organ agar. We hope that others will use this method to quickly perform large screens of other species and find similar success.

## Materials and Methods

### Bacterial strains and plasmids.

*P. mirabilis* strain HI4320 was isolated from the urine of an elderly female nursing home patient with a long-term (≥30 days) indwelling catheter (48, 49). This strain is well established as a model organism for *P. mirabilis* virulence studies, and readily produces UTIs in mice (15, 16). The *P. mirabilis* HI4320 ordered library contains 1728 transposon mutants, each insertion within a different open reading frame, and has been described elsewhere in detail (20). Uropathogenic *Escherichia coli* CFT073 was obtained from a hospitalized patient with pyelonephritis and bacteremia (50). *E. coli* homologs of *P. mirabilis* organ agar hits were identified using BLAST, and selected *E. coli* CFT073 transposon mutants were pulled from a previously reported ordered library (6). Bacteria were routinely cultured at 37°C with aeration in lysogeny broth (LB; 10 g/L tryptone, 5 g/L yeast extract, 0.5 g/L NaCL) or on LB solidified with 1.5% agar. All strains and plasmids are listed in Supplemental Table S2.

### Organ and urine agar.

To make organ agar, five male Swiss Webster mice (outbred, 6–7-week-old, Envigo) were humanely euthanized. To facilitate aseptic organ removal, skin and fur were removed from the abdominal region as previously described prior to opening the abdominal cavity (16). Livers and kidneys were removed, homogenized in 3 ml of water, pooled, and diluted 1:10 in sterile distilled water. 3% agar was autoclaved, cooled to 55°C, and mixed 1:1 with the diluted organ homogenates. This 1:10 dilution was experimentally determined to sustain reliable growth of wild-type *P. mirabilis* HI4320 (Supplemental Fig. S1). Urine agar was made with modifications from a previous protocol (23). Specifically, human urine was collected from seven healthy female volunteers, pooled, and filter sterilized. Aliquots of urine were stored at −20°C. 4% agar buffered with 500 mM HEPES, pH 6.8, was autoclaved, cooled to 55°C, and mixed 1:1 with pooled urine. Both organ and urine agar were precisely aliquoted (25 ml) to 100 mm Petri dishes and allowed to solidify and dry at room temperature overnight.

### Ordered library screen.

Twenty 96-well plates containing the frozen *P. mirabilis* ordered library were placed on dry ice. A sterile 48-pin stamper was used to transfer bacteria onto organ or urine agar plates, which were then incubated at 37°C for 24h or 48h. Plates were visually assessed for decreased or absent growth in each spot. Interesting candidates were rescreened to confirm the phenotype. The genetic location of the transposon was confirmed for a selection of twenty of these mutants using anchored PCR with transposon-specific primer CP-7 and a primer flanking the transposon insertion site (Supplemental Table S3).

### Construction of targeted mutants.

Stable insertional mutations of selected genes were generated using the targetron method (51, 52). Briefly, a group II intron fragment was synthesized (eBlocks, Integrated DNA Technologies) to specifically target each gene using the ClosTron prediction algorithm (53). Reprogrammed intron fragments were cloned into pACD4K-CloxP using NEBuilder HiFi DNA Assembly master mix (New England Biolabs) with primers designed to amplify vector or intron templates and confirmed using Sanger DNA sequencing. Targetron-containing plasmids and a source for T7 polymerase, pAR1219 (54), were introduced into *P. mirabilis* HI4320 using electroporation and induced to jump into the specified genes. Insertional mutations in kanamycin-resistant mutants were confirmed using PCR. Targetron plasmids and mutants are listed in Supplemental Table S2. All primers are listed in Supplemental Table S3.

### Growth curves and chemical complementation of mutants.

Wild-type and mutant *P. mirabilis* were cultured separately overnight in LB, then diluted 1:100 into target media for growth curve analysis using a Bioscreen C (Growth Curves USA). Readings were collected at an optical density of 600 nm (OD_600_) every 15 min for 24 h. Each experiment contained three technical replicates and was conducted at least three times. Minimal A is a minimal, chemically defined medium tailored for *P. mirabilis* and was used for most experiments (55). The carbon source in Minimal A was 0.2% glycerol, unless otherwise specified. For chemical complementation with RNA, RNA purified from *P. mirabilis* HI4320 or isogenic mutants (RNeasy kit, Qiagen) was pooled together, quantified using a NanoDrop spectrophotometer, then added to Minimal A at a final concentration of 15 μg/ml.

### Genetic complementation of mutants.

Complementation plasmid pGEN-MCS, chosen because it is low copy number and is maintained stably without antibiotic selection, was used for genetic complementation of selected mutants (56). Native *P. mirabilis* promoters were predicted using Softberry (57) and included all predicted DNA binding protein sites. Coding sequences and promoters were amplified by PCR, cloned using the Gibson method (New England Biolabs), and constructs were confirmed by Sanger sequencing. Complementation plasmids are listed in Supplemental Table S2 and primers used for cloning are shown in Supplemental Table S3.

### Swarming assays.

Swarming motility was assayed on LB agar containing 10 g/L NaCl as previously described (58). Briefly, strains were cultured overnight in LB (LB 10g/L NaCl), spotted onto swarm agar, and allowed to dry. Swarm plates were incubated at 30°C. Swarm radii were measured after incubation for 19h, and swarms were photographed after incubation for 48h using a Qcount (Advanced Instruments).

### Mouse model of ascending UTI.

Bacterial fitness during UTI was assessed using a well-established mouse model (17, 59, 60). Briefly, overnight cultures of *P. mirabilis* were diluted in LB to OD_600_ = 0.194 (~2 x 10^8^ CFU/ml), then wild-type and mutant bacteria were mixed 1:1. Ten female CBA/J mice, aged 5-6 weeks (Jackson Laboratory), were transurethrally inoculated with 50 μl of this 1:1 mixture (10^7^ CFU/mouse). At 7 days post-inoculation, urine was collected, then mice were euthanized and bladders, kidneys, and spleens were harvested. Organs were homogenized and plated to quantify CFU; mutants were distinguished from wild-type colonies using kanamycin. Competitive indices were calculated for each site by comparing the ratio of output wild type and mutant to the ratio of input bacteria (35). Statistical significance of competitive indices was calculated using the Wilcoxon signed rank test.

### Mouse model bottleneck determination.

Wild-type HI4320 and a kanamycin-resistant mutant that was previously shown to be non-deleterious in our mouse UTI model, *spa47* (51), were mixed in different ratios to test the limitations of our mouse model. The OD_600_ was recorded to normalize strains prior to mixing and adjusted to target ratios. Aliquots of the input mixtures were taken as samples and plated to determine the actual ratio of mutant:wild-type in the inoculum (Fig. 1). The output organ samples collected from CBA/J mice were homogenized and enumerated, via differential plating, to determine mutant and wild-type HI4320 CFU burden. A competitive index calculation was used to demonstrate the output ratio relative to the input ratio (6). A sample that was the same ratio at the beginning and end of the experiment would have Log_10_ CI = 0. The acceptable range of error for these experiments was ±1-log (10-fold) CFU.

### Organ agar for targeted mutants.

Targetron mutants were tested on organ agar made from female CBA/J mice to directly compare with *in vivo* cochallenges in this standard UTI mouse model. Agar was made as above, but in addition to 1:10 dilutions of liver and kidneys, undiluted “1X” organs were used. For bladder and spleen agar, two organs were homogenized together in 3 ml of water to achieve a 2X concentration of each organ. Bacteria were cultured overnight in LB, then adjusted to OD_600_ = 0.1 in LB, aliquoted into a 96-well plate, and stamped onto organ agar. Plates were incubated at 37°C and growth was photographed at 24h and 48h. Mutant PMI2870 was additionally screened on urine and 0.1X liver agar in a 1:10 dilution series to determine whether the amount of inoculum affected growth on organ agar.

### Ethics statement.

Animal experiments were approved by the University of Michigan Medical School Institutional Animal Care and Use Committee, protocol number PRO00007111. During catheterization procedures, mice were anesthetized by intraperitoneal injection of ketamine/xylazine. Mice were euthanized by inhalant isoflurane anesthetic overdose prior to organ removal.

### Statistics and software.

All graphs and charts were plotted and statistics calculated using GraphPad Prism 9. Error bars show SD. To calculate statistical significance for all bar graphs, one-way ANOVA with Dunnett’s multiple comparisons test was used. For mouse cochallenge experiments, statistical significance was calculated using the Wilcoxon signed rank test. *P < 0.05, **P < 0.01, ***P < 0.001, ****P < 0.0001.

## Supplementary Material

1

## Figures and Tables

**Table 1.** Recovery of bacteria from bottleneck experiments

		Number of mice with recovered bacteria		
target ratio	actual ratio	urine	bladder	kidneys
1:1	1:1	5/5	5/5	5/5
1:10	1:8	5/5	5/5	4/5
1:10	1:11	5/5	5/5	4/5
1:25	1:13	5/5	5/5	4/5
1:25	1:36	5/5	5/5	4/5
1:50	1:41	5/5	5/5	3/5
1:50	1:59	10/10	10/10	8/10
1:100	1:88	5/5	5/5	3/5
1:500	1:606	5/5	5/5	4/5
1:1000	1:737	5/5	5/5	5/5
1:1000	1:710	5/5	5/5	4/5

**Table 2.** Organ agar hits

Gene	Name	New Locus Tag	Plate ^a^	Well ^a^	OD_600_	Organ ^b^	Function	General function	Follow-up
PMI0003	*thrC*	PMI_RS00015	216	G4	0.862	U	threonine synthase	Amino acid metabolism	
PMI0205	*hemL*	PMI_RS00985	219	C2	0.500	UK	glutamate-1-semialdehyde 2,1-aminomutase	Amino acid metabolism	
PMI0335	*proC*	PMI_RS01600	202	G2	0.644	U	pyrroline-5-carboxylate reductase	Amino acid metabolism	
PMI0370	*proA*	PMI_RS01770	208	E11	0.727	U	gamma-glutamyl phosphate reductase	Amino acid metabolism	
PMI0711	*serC*	PMI_RS03500	219	C4	0.419	UK	phosphoserine aminotransferase	Amino acid metabolism	Y
PMI1344	*trpD*	PMI_RS06485	204	B8	0.914	K	anthranilate synthase component (glutamine amidotransferase)	Amino acid metabolism	
PMI1348	*trpA*	PMI_RS06505	204	C4	0.889	UK	tryptophan synthase alpha chain	Amino acid metabolism	
PMI2085	*leuB*	PMI_RS10270	207	C5	0.946	U	3-isopropylmalate dehydrogenase	Amino acid metabolism	
PMI2094	*speA*	PMI_RS10315	201	H1	0.571	K	biosynthetic arginine decarboxylase	Amino acid metabolism	
PMI2288	*dapD*	PMI_RS11300	210	E12	0.746	K	2,3,4,5-tetrahydropyridine-2-carboxylate N-succinyltransferase	Amino acid metabolism	
PMI2821	*argD*	PMI_RS13935	210	A4	0.720	U	acetylornithine/succinyldiaminopimelate aminotransferase	Amino acid metabolism	
PMI3027	*aroB*	PMI_RS14975	219	A3	0.734	U	3-dehydroquinate synthase	Amino acid metabolism	
PMI3185	*cysE*	PMI_RS15750	212	G7	0.954	UL	serine acetyltransferase	Amino acid metabolism	
PMI3236	*argE*	PMI_RS16000	209	F6	0.812	U	acetylornithine deacetylase	Amino acid metabolism	
PMI3301	*ilvE*	PMI_RS16410	207	B4	1.016	U	branched-chain amino acid aminotransferase	Amino acid metabolism	
PMI3302	*ilvD*	PMI_RS16415	207	D2	0.913	U	dihydroxy-acid dehydratase	Amino acid metabolism	
PMI3457	*argI*	PMI_RS17230	204	D1	0.911	UL	ornithine carbamoyltransferase chain I	Amino acid metabolism	Y
PMI3528	*metR*	PMI_RS17535	201	A2	0.728	U	LysR-family transcriptional regulator	Amino acid metabolism	
PMI0006	*talB*	PMI_RS00030	213	G2	0.818	UK	transaldolase B	Carbohydrate metabolism	
PMI3319	*rffD*	PMI_RS16495	219	C5	0.398	K	UDP-N-acetyl-D-mannosamine dehydrogenase	Carbohydrate metabolism	
PMI3180	*envC*	PMI_RS15725	211	E2	0.564	K	putative exported peptidase/ murein hydrolase activator EnvC	Cell division	
PMI3384	*fklB*	PMI_RS16845	213	E11	0.588	K	FkbP-type 22 kDa peptidyl-prolyl cis-trans isomerase	Chaperones and folding catalysts	
PMI1912		PMI_RS09435	207	F3	0.737	U	FtsK/SpoIIIE-family protein	Chromosome partitioning	
PMI3538	*ubiB*	PMI_RS17585	206	A11	0.939	U	probable ubiquinone biosynthesis protein	Cofactor metabolism	
PMI2309	*recB*	PMI_RS11420	219	B3	0.241	UK	exodeoxyribonuclease V beta chain	DNA repair	
PMI2930	*glpD/glyD*	PMI_RS14485	210	D8	0.727	K	aerobic glycerol-3-phosphate dehydrogenase	Lipid metabolism	
PMI3210	*glpK*	PMI_RS15875	214	E2	0.958	U	glycerol kinase	Lipid metabolism	Y
PMI3175	*waaF/rfaF*	PMI_RS15700	219	B2	0.402	K	ADP-heptose--LPS heptosyltransferase II	Lipopolysaccharide biosynthesis	
PMI3316	*wecA/rfe*	PMI_RS16480	219	D6	0.351	U	undecaprenyl-phosphate alpha-N-acetylglucosaminyl 1-phosphate transferase	Lipopolysaccharide biosynthesis	
PMI1545	*guaA*	PMI_RS07520	201	D12	0.807	UKL	GMP synthase [glutamine-hydrolyzing]	Nucleotide metabolism	Y
PMI1562	*purC*	PMI_RS07610	209	G4	0.880	U	phosphoribosylaminoimidazole-succinocarboxamide synthetase (SAICAR	Nucleotide metabolism	
PMI1875	*purL*	PMI_RS09255	201	C11	0.737	U	phosphoribosylformylglycineamide synthetase	Nucleotide metabolism	
PMI3028		PMI_RS14980	213	F2	0.946	U	SPOR domain-containing protein	Peptidoglycan binding	
PMI0200	*dksA*	PMI_RS00965	211	F9	0.877	K	DnaK suppressor protein	rRNA transcription factor	
PMI0765	*ompF/nmpC*	PMI_RS03760	218	G6	0.716	UK	outer membrane porin	Signal transduction	
PMI3431		PMI_RS17100	213	E9	0.974	U	two-component system sensor kinase	Signaling and cellular processes	
PMI0570	*sucB*	PMI_RS02805	207	F6	0.863	UKL	dihydrolipoamide succinyltransferase component of 2-oxoglutarate dehydrogenase complex	TCA cycle ^c^	Y
PMI0083	*nusB*	PMI_RS00400	210	C11	0.333	K	N utilization substance protein B	Transcription machinery	
PMIt068		PMI_RS16125	219	C1	0.490	K	tRNA	Transcription machinery	
PMI1151	*znuC*	PMI_RS05555	201	G4	0.775	KL	high-affinity zinc uptake system ATP-binding protein	Transport	
PMI1152	*znuA*	PMI_RS05560	209	C1	0.990	K	high-affinity zinc uptake system substrate-binding protein	Transport	
PMI1833	*cysW*	PMI_RS09045	201	A5	0.609	K	sulfate/thiosulfate ABC transporter, permease protein	Transport	
PMI1945	*ireA*	PMI_RS09585	202	A9	0.714	U	putative TonB-dependent ferric siderophore receptor	Transport	
PMI0005		PMI_RS00025	213	F6	0.880	U	conserved hypothetical protein		
PMI0206	*erpA*	PMI_RS00990	211	E9	0.576	UK	putative iron-sulfur protein		
PMI0641	*sanA*	PMI_RS03160	215	C10	0.854	K	putative transport protein		Y
PMI2720		PMI_RS13405	213	D5	0.771	U	BMC domain-containing protein		
PMI2870		PMI_RS14185	208	F4	0.575	UKL	hypothetical protein		Y

a location of transposon mutants in the ordered library

b Urine, Kidney, Liver

c *sucB*, primarily known as a component of the TCA cycle, is also involved in amino acid metabolism; it was included as a metabolic gene for purposes of categorization in Fig. 2.
