# Supplementary material for: Organ agar serves as physiologically relevant alternative for in vivo colonization
Source: Res Sq. 2023 May 19:rs.3.rs-2777869. Preprint. [Version 1] doi: 10.21203/rs.3.rs-2777869/v1 (PMC10246091; doi:10.21203/rs.3.rs-2777869/v1)
Supplement: 1 [file NIHPPrs2777869v1-supplement-1.pdf]

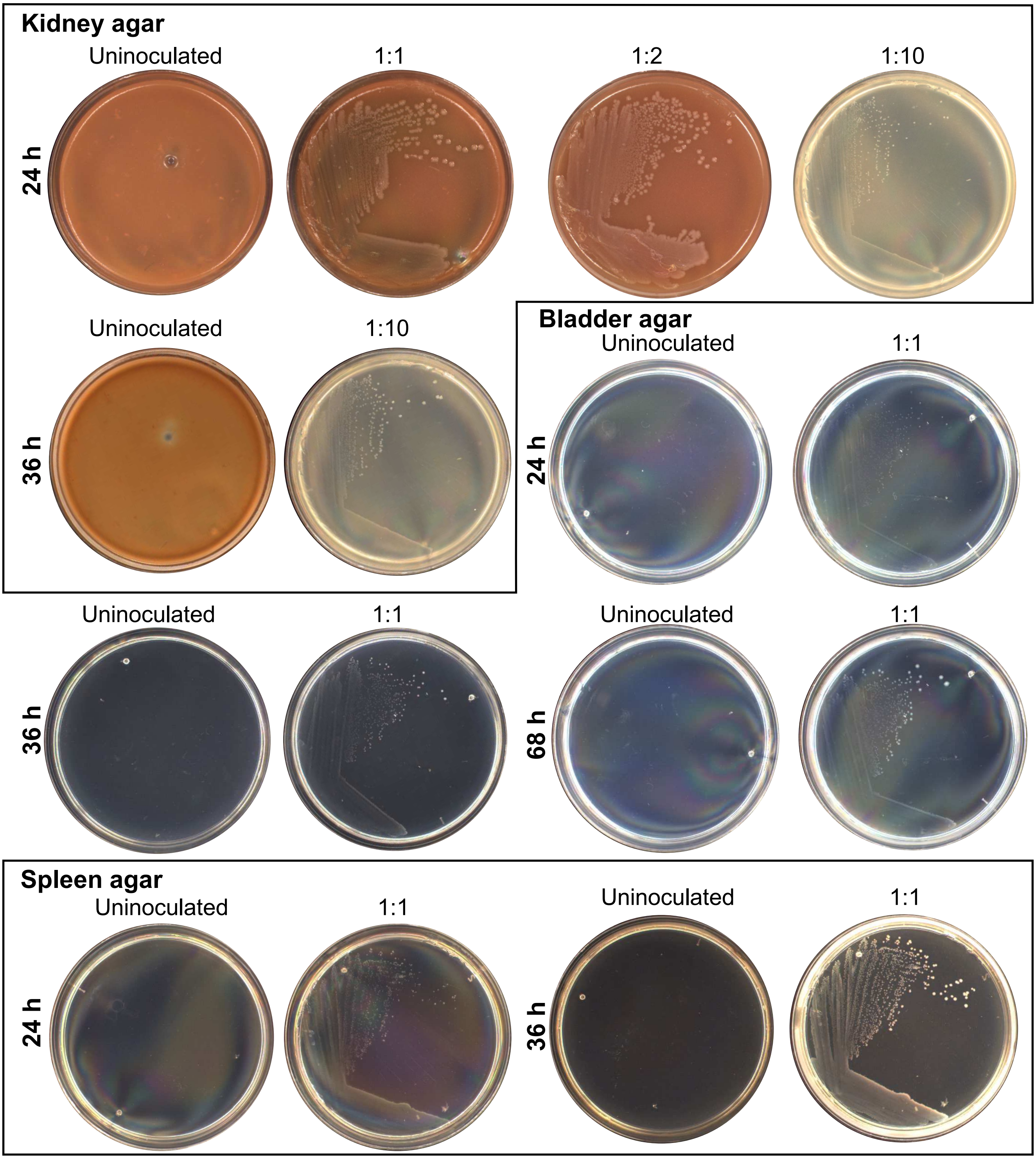

Supplemental Fig. S1. Optimizing organ agar. A single colony of *P. mirabilis* HI4320 was streaked on organ agars and incubated at 37°C for the indicated times. On kidney agar, colonies were visible within 24 h up to a 1:10 dilution, whereas colonies on bladder or spleen agar were only visible on undiluted agar, and became more visible following longer incubation. Thus, larger organs such as kidneys or livers facilitate larger screens with fewer mice.

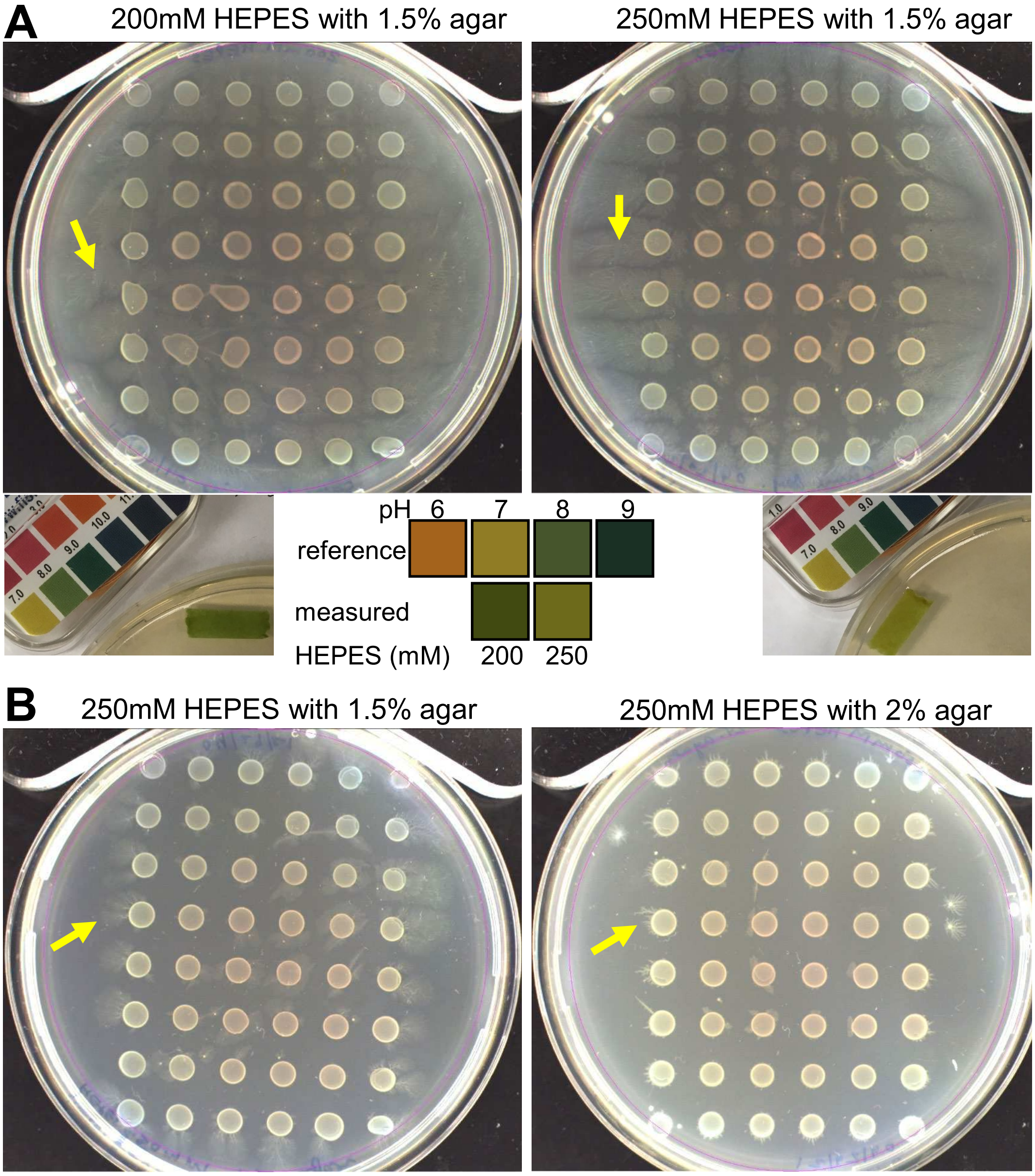

Supplemental Fig. S2. Optimizing urine agar. Wild-type *P. mirabilis* HI4320 was stamped onto agar made from human urine and incubated at 37°C for 24 h. **(A)** Comparison of buffering capacity. Urine agar was buffered with either 200 mM or 250 mM HEPES (pH 6.8), and pH paper placed on the agar surface after incubation was used to gauge the agar pH of each plate. 200 mM HEPES was insufficient to hold the pH below 8. Swarming motility from the points of inoculation is visible on both plates (arrows). **(B)** Comparison of agar concentration. Increasing the agar concentration from 1.5 to 2% greatly reduced swarming motility (arrows).

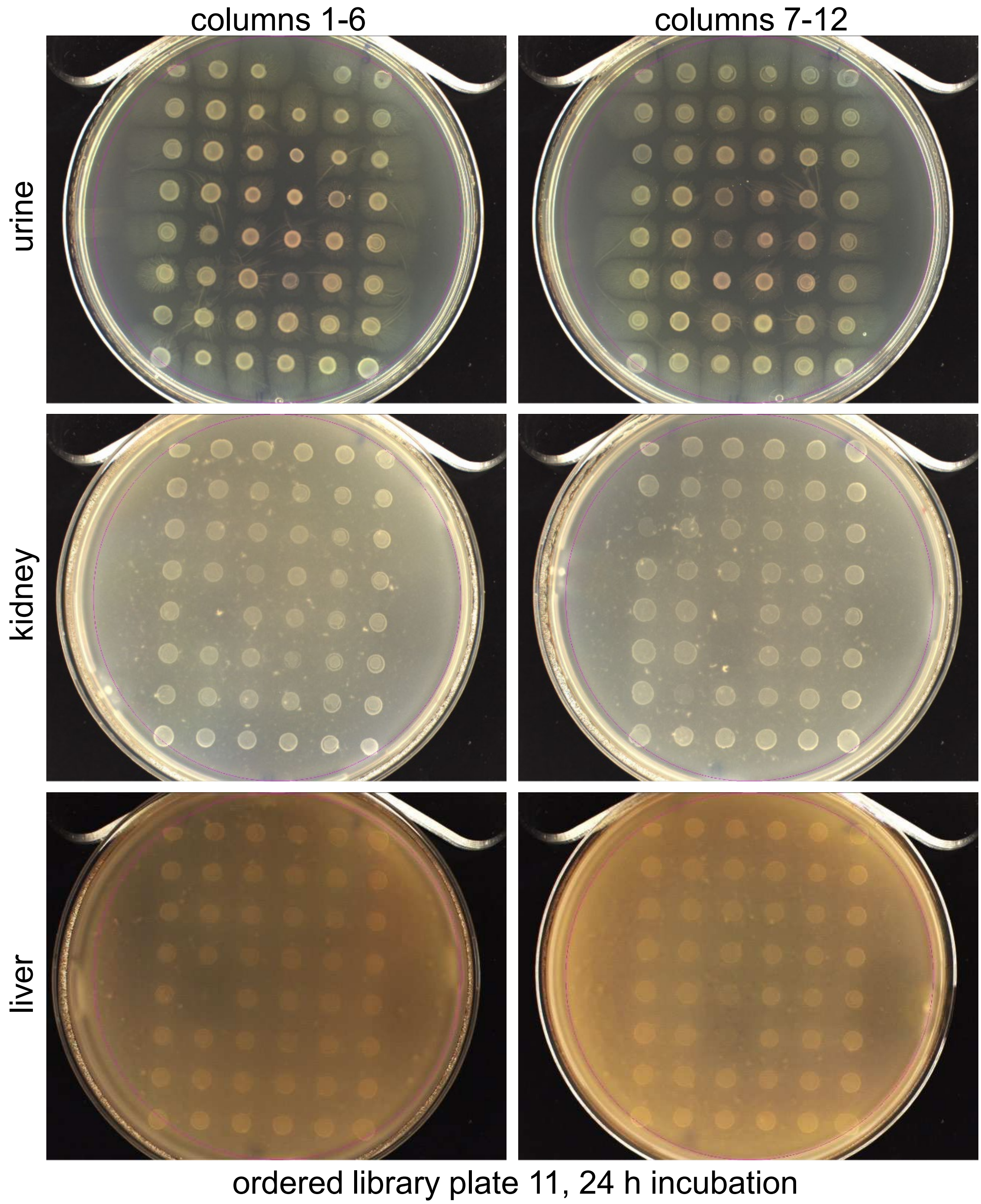

Supplemental Fig. S3. Example of organ agar screening from ordered transposon library. The screen of plate 11 from the ordered library is shown here at 24 hpi. Each Petri dish accommodates 48 samples; thus, two agar plates per organ were required to screen one 96-well plate of transposon mutants. Mutants with differential growth on urine, kidney, or liver agars can be seen.

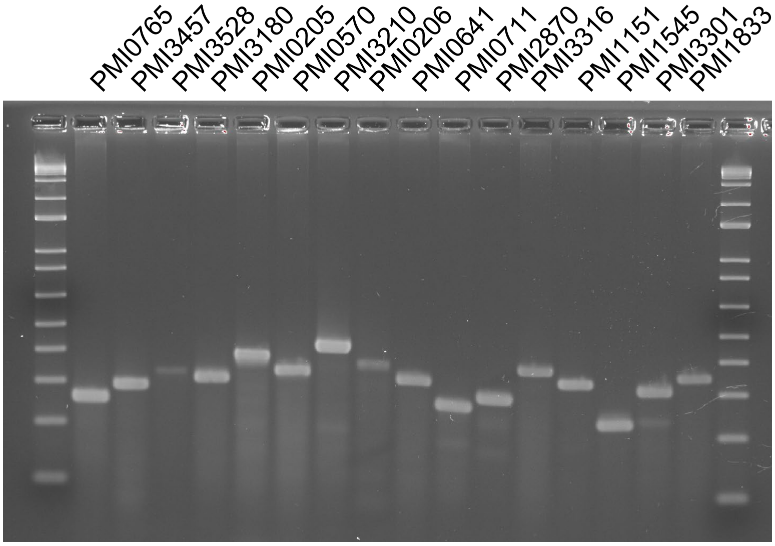

Supplemental Fig. S4. Confirmation of transposon insertions in ordered library. PCR using transposon-specific primer CP-7 and gene-specific primers was conducted to confirm the identify of transposon mutants in the *P. mirabilis* ordered library. The predicted insertion was confirmed for 16/20 mutants.

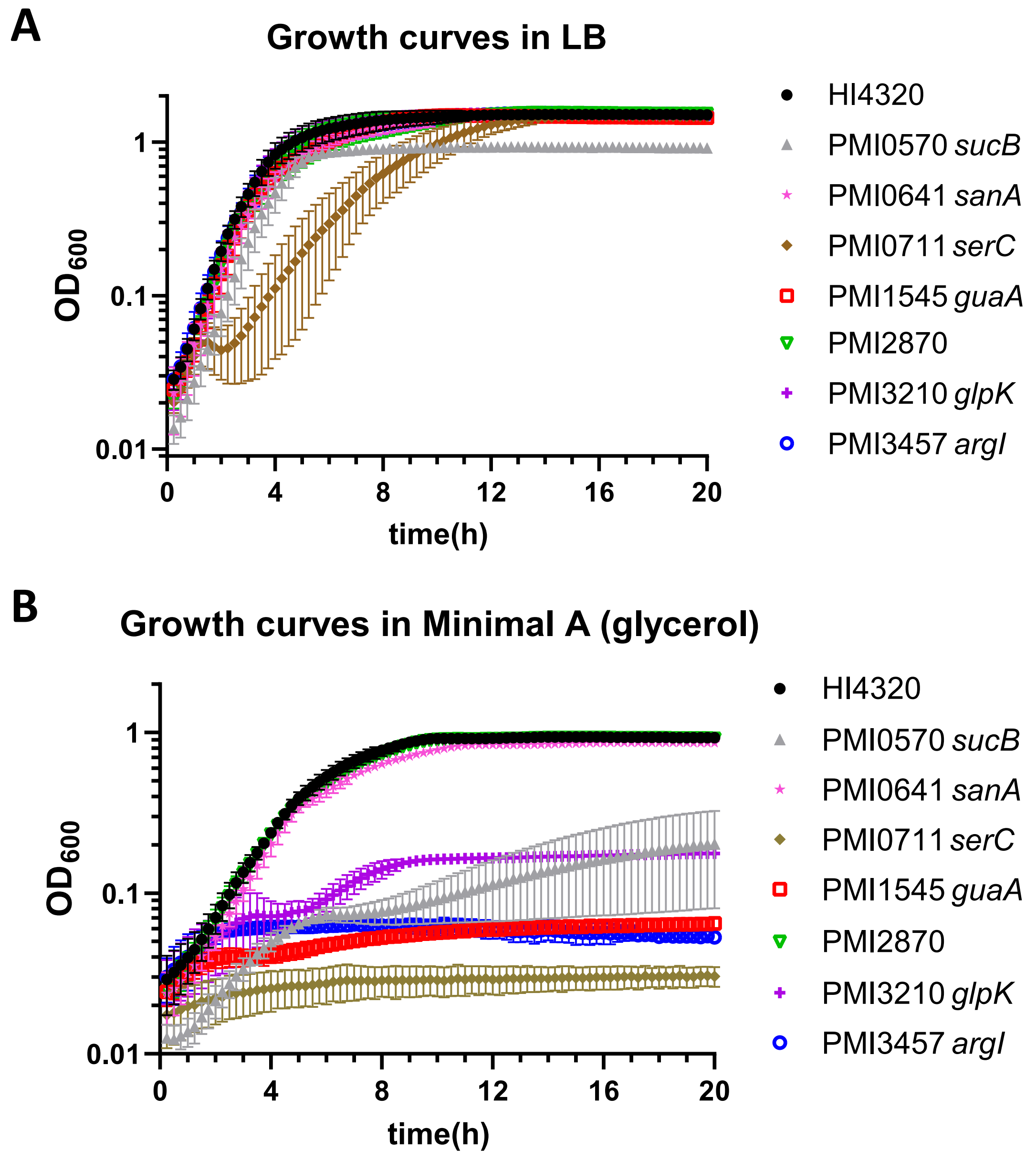

Supplemental Fig. S5. Growth curves for wild-type HI4320 and mutants. **(A)** LB; **(B)** Minimal A. These curves are the same data used to generate the area under the curve results shown in Fig. 3. Error bars show SD.

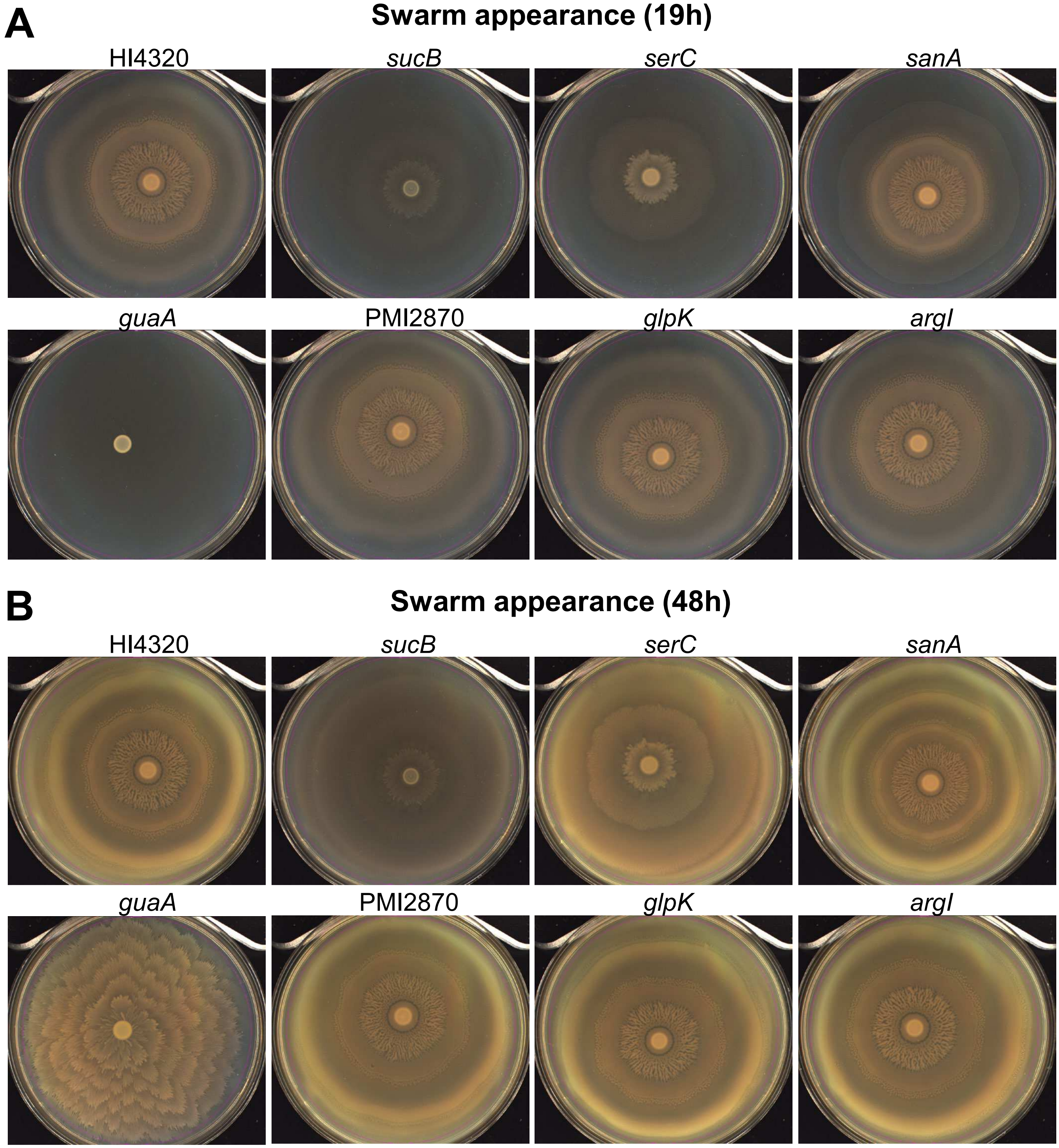

Supplemental Fig. S6. Swarming by *P. mirabilis* mutants. **(A)** Swarming after 19h at 30°C, before wild-type swarms reached the edge of the agar surface. **(B)** Swarming after 48h incubation at 30°C, when swarming and consolidation rings are more visible.

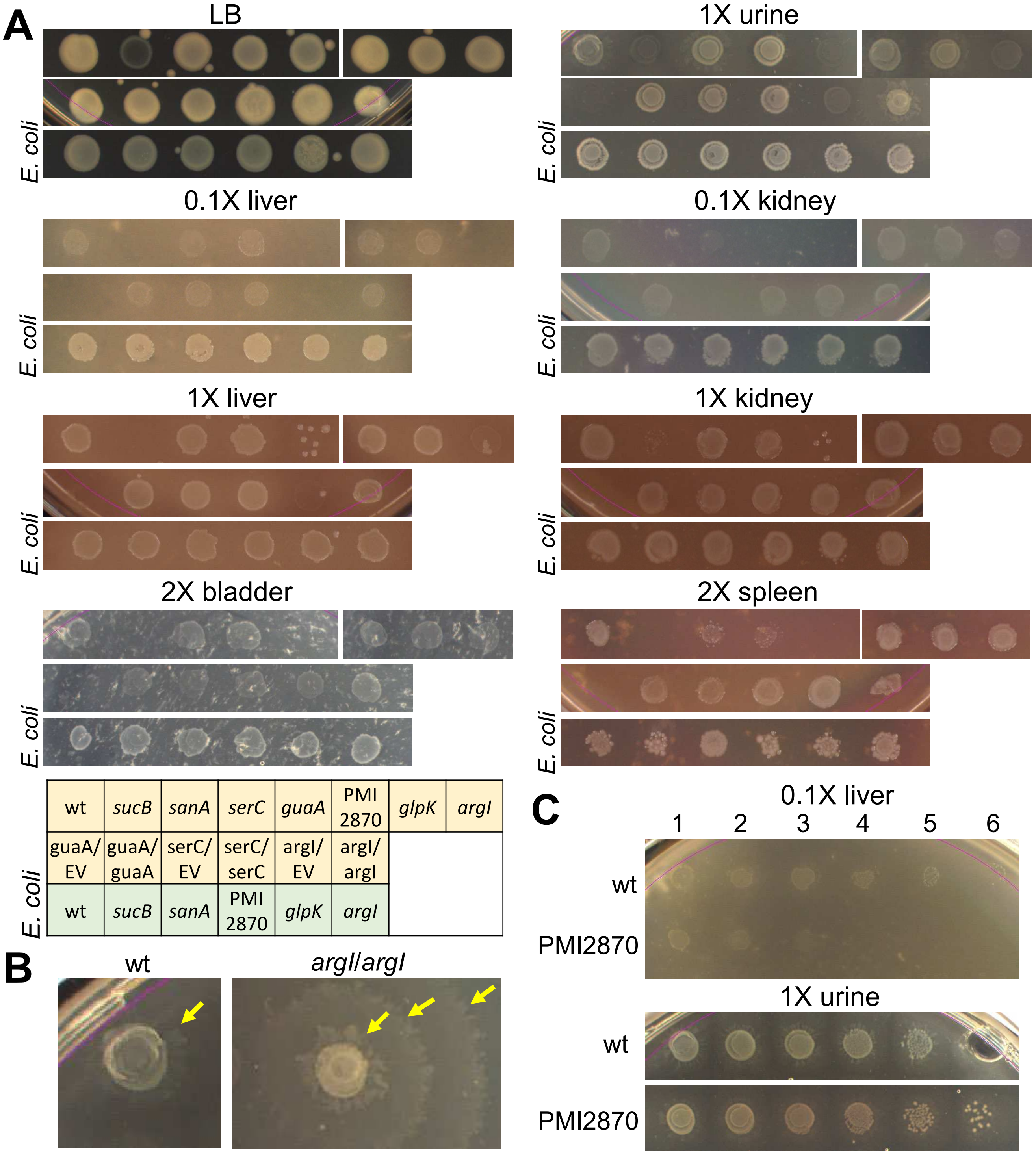

Supplemental Fig. S7. Organ agar made from UTI model mice gives similar results as agar made from outbred Swiss-Webster mice. (A) Targetron mutants were cultured in LB medium, adjusted to $OD_{600} = 0.1$, then stamped onto organ agar made from female CBA/J mice or agar made from pooled human urine. Photos were taken after 24 h incubation at 37°C. Homologous mutations in uropathogenic *E. coli* CFT073 pulled from an ordered transposon library were also tested, when available. Strains are indicated in the schematic at the bottom, with *P. mirabilis* in yellow and *E. coli* in green. **(B)** Magnification of wild-type *P. mirabilis* and the *argI* mutant complemented with *argI* on urine agar. Swarm rings are indicated with yellow arrows. **(C)** Dilution series on 1:10 liver agar showing growth defect for PMI2870 that is only visible when fewer bacteria were stamped. Column 1 was stamped from LB broth cultures that had been adjusted to $OD_{600} = 1.0$. Columns 2-6 are serial 1:10 dilutions. A similar experiment on urine agar did not reveal a defect for this mutant in a dilution series.

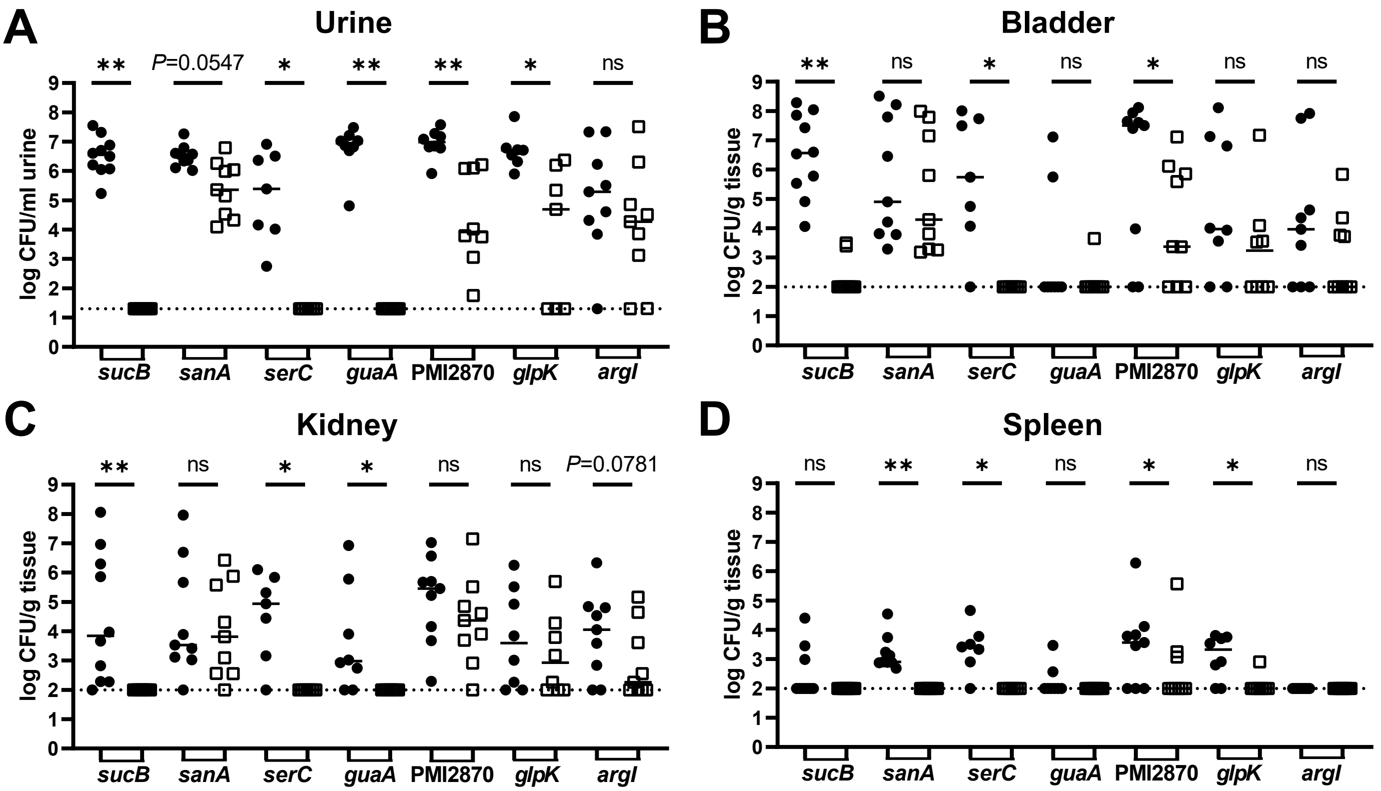

Supplemental Fig. 8. Total bacterial recovery from murine cochallenge experiment shown in Fig. 6. Female CBA/J mice were transurethrally inoculated with a 1:1 mixture of HI4320 (wt) and the indicated mutant. After 7 days, urine was collected, then organs were collected, homogenized, and plated to enumerate bacterial burden. Dotted lines show the limit of detection (for urine, 20; for organ homogenates, 100). *P* values were assessed using the Wilcoxon signed rank test (**P*<0.05; ***P*<0.01; for *P* values between 0.1 and 0.05, the exact value is given; ns, not significant). ●, wt; □, mutant.

Supplemental Table S1. Organ agar hits selected for PCR validation

Gene	Name	New Locus Tag	Function	Organ hit	Valid
PMI0006	*talB*	PMI_RS00030	transaldolase B	UK	No
PMI0205	*hemL*	PMI_RS00985	glutamate-1-semialdehyde 2,1-aminomutase	UK	Yes
PMI0206	*erpA*	PMI_RS00990	putative iron-sulfur protein	UK	Yes
PMI0570	*sucB*	PMI_RS02805	dihydrolipoamide succinyltransferase component of 2-oxoglutarate dehydrogenase complex	UKL	Yes
PMI0641	*sanA*	PMI_RS03160	putative transport protein	K	Yes
PMI0711	*serC*	PMI_RS03500	phosphoserine aminotransferase	UK	Yes
PMI0765	*ompF*	PMI_RS03760	outer membrane porin	UK	Yes
PMI1151	*znuC*	PMI_RS05555	high-affinity zinc uptake system ATP-binding protein	KL	Yes
PMI1545	*guaA*	PMI_RS07520	GMP synthase [glutamine-hydrolyzing]	UKL	Yes
PMI1833	*cysW*	PMI_RS09045	sulfate/thiosulfate ABC transporter, permease protein	K	Yes
PMI2870		PMI_RS14185	hypothetical protein	UKL	Yes
PMI3180	*envC*	PMI_RS15725	putative exported peptidase/ murein hydrolase activator EnvC	K	Yes
PMI3210	*glpK*	PMI_RS15875	glycerol kinase	U	Yes
PMI3301	*ilvE*	PMI_RS16410	branched-chain amino acid aminotransferase	U	Yes
PMI3316	*wecA/rfe*	PMI_RS16480	undecaprenyl-phosphate alpha-N-acetylglucosaminyl 1-phosphate transferase	U	Yes
PMI3384	*fklB*	PMI_RS16845	FkbP-type 22 kDa peptidyl-prolyl cis-trans isomerase	K	No
PMI3431		PMI_RS17100	two-component system sensor kinase	U	No
PMI3457	*argI*	PMI_RS17230	ornithine carbamoyltransferase chain I	UL	Yes
PMI3528	*metR*	PMI_RS17535	LysR-family transcriptional regulator	U	Yes
PMI3538	*ubiB*	PMI_RS17585	probable ubiquinone biosynthesis protein	U	No

Supplemental Table S2. Bacterial strains and plasmids

Bacterial strains used in this study

Name	Selection	Description	Reference
HI4320	tet	Wild-type clinical isolate of *P. mirabilis*	1
Top 10		Routine *E. coli* host for cloning	Invitrogen
PMI0570::kan	kan	*sucB* targetron mutant	This study
PMI0641::kan	kan	*sanA* targetron mutant	This study
PMI0711::kan	kan	*serC* targetron mutant	This study
PMI1545::kan	kan	*guaA* targetron mutant	This study
PMI2870::kan	kan	PMI2870 targetron mutant	This study
PMI3210::kan	kan	*glpK* targetron mutant	This study
PMI3457::kan	kan	*argI* targetron mutant	This study
CFT073		Wild-type clinical isolate of *E. coli*	
CFT073 *sucB*::Tn	kan	*sucB* transposon mutant from ordered library	2
CFT073 *sanA*::Tn	kan	*sanA* transposon mutant from ordered library	2
CFT073 *yicH*::Tn	kan	*PMI2870* transposon mutant from ordered library	2
CFT073 *glpK*::Tn	kan	*glpK* transposon mutant from ordered library	2
CFT073 *argI*::Tn	kan	*argI* transposon mutant from ordered library	2

Plasmids used in this study

Name	Selection	Description	Reference
pAR1219	amp	T7 helper plasmid for targetron mutagenesis	3
pACD4K-CloxP-lacZ	chl	Targetron mutagenesis vector	Millipore Sigma
pSNS-2A1	chl	pACD4K-CloxP with intron targeting PMI3457 *argI*	This study
pSNS-2A2	chl	pACD4K-CloxP with intron targeting PMI0570 *sucB*	This study
pSNS-2A3	chl	pACD4K-CloxP with intron targeting PMI2870	This study
pSNS-2A4	chl	pACD4K-CloxP with intron targeting PMI3210 *glpK*	This study
pSNS-2A5	chl	pACD4K-CloxP with intron targeting PMI0641 *sanA*	This study
pSNS-2A7	chl	pACD4K-CloxP with intron targeting PMI0711 *serC*	This study
pSNS-2A8	chl	pACD4K-CloxP with intron targeting PMI1545 *guaA*	This study
pGEN-MCS	amp	Complementation plasmid; stably maintained without selection	4
guaA	amp	promoter	This study
pGEN-serC	amp	Complementation of *serC* under its native promoter	This study
pGEN-argI	amp	Complementation of *argI* under its native promoter	This study

References

1. Mobley HLT, Warren JW. Urease-positive bacteriuria and obstruction of long-term urinary catheters. J Clin Microbiol. 1987;25:2216-7.

2. Shea AE, Marzoa J, Himpsl SD, Smith SN, Zhao L, Tran L, Mobley HLT. *Escherichia coli* CFT073 fitness factors during urinary tract infection: identification using an ordered transposon library. Appl Environ Microbiol. 2020;86(13). Epub 2020/05/03. doi: 10.1128/AEM.00691-20. PubMed PMID: 32358013; PMCID: PMC7301846.

3. Davanloo P, Rosenberg AH, Dunn JJ, Studier FW. Cloning and expression of the gene for bacteriophage T7 RNA polymerase. Proc Natl Acad Sci U S A. 1984;81(7):2035-9. Epub 1984/04/01. doi: 10.1073/pnas.81.7.2035. PubMed PMID: 6371808; PMCID: PMC345431.

4. Lane MC, Alteri CJ, Smith SN, Mobley HLT. Expression of flagella is coincident with uropathogenic *Escherichia coli* ascension to the upper urinary tract. Proc Natl Acad Sci U S A. 2007;104(42):16669-74.

Supplemental Table S3. Primers used in this study

Name	Sequence	Purpose
CP-7	CCAAGCAGAAGACGGCATACG	transposon anchored primer for confirmation of insertions
PMI0205 Rev	AGTTTGGTTGTCCCATCGTC	PCR confirmation of transposon insertion in PMI0205, paired with CP-7
PMI0206 Rev	AGATGTGGGGATTGCTAACG	PCR confirmation of transposon insertion in PMI0206, paired with CP-7
PMI0570 Rev	CGTCGTATCTTGTGCAGGTT	PCR confirmation of transposon insertion in PMI0570, paired with CP-7
PMI0641 Rev	TCCATAACGATACGCGATGC	PCR confirmation of transposon insertion in PMI0641, paired with CP-7
PMI0711 Rev	GCCACCATGACAGAACAGAA	PCR confirmation of transposon insertion in PMI0711, paired with CP-7
PMI0765 Rev	TCAAAACGACCGAAACCAGT	PCR confirmation of transposon insertion in PMI0765, paired with CP-7
PMI1151 Fwd	CCGCAATAAACCCTATCGGC	PCR confirmation of transposon insertion in PMI1151, paired with CP-7
PMI1545 Fwd	GCCGGTATTAGGCATCTGCT	PCR confirmation of transposon insertion in PMI1545, paired with CP-7
PMI1833 Fwd	ATTGCATGGCAGACCGAAGT	PCR confirmation of transposon insertion in PMI1833, paired with CP-7
PMI2870 Rev	GTCTTTGTCTCGCTGTTCCA	PCR confirmation of transposon insertion in PMI2870, paired with CP-7
PMI3180 Rev	TTTGTAAGCGGTTTTGCGTC	PCR confirmation of transposon insertion in PMI3180, paired with CP-7
PMI3210 Rev	GGCACCACTTTAGGTAGCAT	PCR confirmation of transposon insertion in PMI3210, paired with CP-7
PMI3301 Fwd	GTGAGCAGTCAACGACCTGT	PCR confirmation of transposon insertion in PMI3301, paired with CP-7
PMI3316 Rev	CACCAAACGAAACGCAAGAA	PCR confirmation of transposon insertion in PMI3316, paired with CP-7
PMI3457 Rev	AAGGATTTGCTAAGGTCGCC	PCR confirmation of transposon insertion in PMI3457, paired with CP-7
PMI3528 Rev	GGTAGATGCTGGAACCCTTG	PCR confirmation of transposon insertion in PMI3528, paired with CP-7
PMI0006 Fwd	AGCAGGTTGTGATCGCCTAA	PCR-confirmed lack of transposon insertion in PMI0006
PMI0006 Rev	ATCGCAGGTAAAGAGAAGGC	PCR-confirmed lack of transposon insertion in PMI0006
PMI3384 Fwd	CAAGACGGGAGGTTTACGCT	PCR-confirmed lack of transposon insertion in PMI3384
PMI3384 Rev	ACCACGCTGTACTGAGCTAT	PCR-confirmed lack of transposon insertion in PMI3384
PMI3431 Fwd	CGCGGAAAAGAACGGCTAATG	PCR-confirmed lack of transposon insertion in PMI3431
PMI3431 Rev	GAGGGAATAGCGGTGTAAGC	PCR-confirmed lack of transposon insertion in PMI3431
PMI3538 Fwd	CCATTTTTACCCGATGGGCG	PCR-confirmed lack of transposon insertion in PMI3538
PMI3538 Rev	TCAACGTGTAACTCAGCCAC	PCR-confirmed lack of transposon insertion in PMI3538
pACD4K-C-Gib-fwd	TAGGAGAACCTATGGGAAC	Amplification of targetron plasmid pACD4K-CloxP for Gibson cloning
pACD4K-C-Gib-rev	CTAGAGGGGAATTGTTATC	Amplification of targetron plasmid pACD4K-CloxP for Gibson cloning
intron-Gib-fwd	ggataacaattcccctctagAAAAAAGCTTATAATTATCCTTA	Amplification of targetron synthetic intron fragment for Gibson cloning
intron-Gib-rev	cgttcccataggttctcctaCAGATTGTACAAATGTGGTG	Amplification of targetron synthetic intron fragment for Gibson cloning
PMI0570-TTF	TTGTGTTAGAAGTACCGGCG	PCR confirmation of targetron insertion in PMI0570 *sucB*
PMI0570-TTR	GTCGGGCTAAGAGCATCATT	PCR confirmation of targetron insertion in PMI0570 *sucB*
PMI0641-TTF	GTTTGGGACAGATGGCTTTAC	PCR confirmation of targetron insertion in PMI0641 *sanA*
PMI0641-TTR	CTAGAAAACGCGGTTCACG	PCR confirmation of targetron insertion in PMI0641 *sanA*
PMI0711-TTF	TAGGTGAGAAAACCACCGC	PCR confirmation of targetron insertion in PMI0711 *serC*

PMI0711-TTR	AGCAATCCCATCGATAGTCTC	PCR confirmation of targetron insertion in PMI0711 *serC*
PMI1545-TTF	CGAGAAAGGCTTGAAAACAGA	PCR confirmation of targetron insertion in PMI1545 *guaA*
PMI1545-TTR	TCTTCGGTAACATCCCATGC	PCR confirmation of targetron insertion in PMI1545 *guaA*
PMI2870-TTF	CAATGGGGTGCGCAAAAA	PCR confirmation of targetron insertion in PMI2870
PMI2870-TTR	CAGATGAGACGCCGTAGTAA	PCR confirmation of targetron insertion in PMI2870
PMI3210-TTF	TGAAACGACTATCGTCTGGG	PCR confirmation of targetron insertion in PMI3210 *glpK*
PMI3210-TTR	CGCCTTCAACATTATCGAGG	PCR confirmation of targetron insertion in PMI3210 *glpK*
PMI3457-TTF	AAAAGATTCCACCCGTACCC	PCR confirmation of targetron insertion in PMI3457 *argI*
PMI3457-TTR	TCAGCATATTTTGCCAGCAT	PCR confirmation of targetron insertion in PMI3457 *argI*
pGEN-GibsonF	aagatcccaacgaaaagc	Amplification of complementation plasmid pGEN-MCS for Gibson cloning
pGEN-GibsonR	caagcttcatatgcccgg	Amplification of complementation plasmid pGEN-MCS for Gibson cloning
PguaBA-GibF	ccccgggcatatgaagcttgatcaataaaaattttcaaaaaaagctag	Amplification of *guaBA* promoter for Gibson cloning
PguaBA-GibR	ttgctgtcatagcaatatctcaccaagg	Amplification of *guaBA* promoter for Gibson cloning
guaA-GibF	agatattgctatgacagcaaatatccataatc	Amplification of *guaA* gene for Gibson cloning
guaA-GibR	acgcttttcgttgggatcttttattcccactcaatcgttg	Amplification of *guaA* gene for Gibson cloning
serC_GibF	ccccgggcatatgaagcttggtttacaaaagtcattgcgccaattag	Amplification of *serC* and promoter for Gibson cloning
serC_GibR	acgcttttcgttgggatcttttaagcgtggcgacgctc	Amplification of *serC* and promoter for Gibson cloning
argI_GibF	ccccgggcatatgaagcttgcaaaagtgacgtttaagg	Amplification of *argI* and promoter for Gibson cloning
argI_GibR	acgcttttcgttgggatcttttaaaaaggatttgctaaggtc	Amplification of *argI* and promoter for Gibson cloning

Supplementary Files

This is a list of supplementary files associated with this preprint. Click to download.

- 246hswarmcomparison.gif
